# Shortened Pulmonary Pulse Wave Transit Time in High-Altitude Residents Without Pulmonary Hypertension: A Doppler Echocardiography Study

**DOI:** 10.33549/physiolres.935663

**Published:** 2026-04-01

**Authors:** Donghua WANG, Yapeng LI, Hongye WANG, Jie ZHANG, Xuchu WU, Xiaozhi ZHENG

**Affiliations:** 1 Department of Ultrasound, Minhang Hospital, Fudan University, Shanghai, China; 2 Department of Ultrasound, Yangpu Hospital, School of Medicine, Tongji University, Shanghai, China; 3 Department of Ultrasound, The Affiliated Lianyungang Hospital of Xuzhou Medical University/The First People’s Hospital of Lianyungang, Lianyungang, Jiangsu Province, China

**Keywords:** Pulmonary pulse wave transit time, High-altitude, Pulmonary hypertension, Echocardiography

## Abstract

Pulmonary pulse wave transit time (pPTT) in high-altitude residents without pulmonary hypertension had not been well characterized. The purpose of this study was to investigate alterations in pPTT among high-altitude residents through comparative analysis with low-altitude counterparts. Between October 2024 and June 2025, 47 consecutive high-altitude (≥ 4000 meters above sea level) residents without pulmonary hypertension were enrolled in this study. pPTT was quantified by Doppler echocardiography. The factors related to pPTT were determined by multiple linear regression analysis. High-altitude residents exhibited significantly larger right cardiac dimension and function, but significantly lower left ventricular mass index and ejection fraction (*p* < 0.05). Notably, pPTT was markedly shorter in high-altitude residents compared to their low-altitude counterparts (144.00 [109.00–193.00] vs. 193.50 [179.00–285.00] ms, *p* < 0.0001). Furthermore, among high-altitude residents, males had significantly shorter pPTT than females (123.00 [102.00–162.00] ms vs. 181.50 [148.00–241.00] ms, *p* < 0.0001). Stepwise multiple regression analysis indicated that altitude was the independent determinant of pPTT in high-altitude residents (*p* < 0.05). The observed reduction in pPTT among high-altitude dwellers without pulmonary hypertension suggests adaptive vascular changes—likely mediated by hypoxia-driven arterial remodeling and elastin degradation. These findings position pPTT as a promising indicator for detecting subclinical pulmonary vascular adaptations in chronic hypoxic environments.

## Introduction

Indigenous populations inhabiting high-altitude regions (≥ 2,500m above sea level) experience chronic exposure to hypobaric hypoxia. Intriguingly, the majority develop intricate adaptive mechanisms that maintain normal pulmonary arterial pressure without progressing to pathological pulmonary hypertension. This distinctive physiological adaptation implies that significant functional remodeling likely occurs in the pulmonary vasculature of healthy high-altitude dwellers. Current research on pulmonary artery function in high-altitude populations primarily relies on traditional hemodynamic parameters such as pulmonary artery systolic pressure (PASP) and pulmonary vascular resistance. However, these indicators typically only detect abnormalities after significant vascular functional changes have occurred, making them inadequate for reflecting early adaptive alterations.

Pulmonary pulse wave transit time (pPTT), defined as the time it takes for the systolic pressure pulse wave to travel from the pulmonary valve to the pulmonary veins, has been recognized as the first sign and a promising surrogate marker of pulmonary hemodynamic and vascular alterations in pulmonary hypertension (PAH) [1–3], chronic obstructive pulmonary disease [4], pulmonary fibrosis [3], systemic lupus erythematosus [5], systemic sclerosis [6] and rheumatoid arthritis [7] due to its non-invasive nature and excellent reproducibility. Clinical studies have demonstrated a marked shortening of pPTT in these patients, with strong correlations to disease severity. However, existing pPTT researches have primarily focused on cases in low-altitude populations, while its behavior in high-altitude residents - particularly those without PAH - remains unexplored.

This study aims to address two questions by comparing pPTT between high-altitude residents without PAH and their low-altitude counterparts: (i) whether chronic high-altitude exposure alters pPTT, and (ii) what factors determine pPTT characteristics. The findings will provide critical insights into adaptive remodeling mechanisms of pulmonary vasculature under hypoxic conditions and potentially establish pPTT as a novel screening tool for early-stage pulmonary vascular disorders in high-altitude populations.

## Methods

### Study population

This study was approved by the Human Research Ethics Committee of Yangpu Hospital, School of Medicine, Tongji University (No. LL-2024-SHZRKX-001). Written informed consent was provided by all participants prior to enrollment. The study was conducted in accordance with the Declaration of Helsinki (as revised in 2013). Between October 2024 and June 2025, consecutive high-altitude residents (≥ 4000 meters above sea level) with ≥ 3 consecutive generations of altitude residence who had no symptomatic complaints or detectable physical signs, were enrolled in this study. The low-altitude control subjects were matched to the high-altitude group not only for age, sex, but also for body surface area (BSA) and key lifestyle factors. Specifically, through questionnaire verification, the vast majority of participants in both groups were engaged in outdoor manual labor (primarily as farmers), ensuring comparability in terms of physical activity levels and occupational exposure to minimize the potential confounding effects of these factors on our results. All the participants underwent comprehensive diagnostic evaluation comprising: (1) systematic review of cardiopulmonary history, (2) thorough physical examination, (3) laboratory investigations (including tumor markers and myocardial enzymes), (4) electrocardiography, and (5) multimodality imaging (radiography, ultrasonography, and computed tomography). Exclusion criteria encompassed: (i) confirmed cardiopulmonary or systemic pathologies (hypertension, pulmonary hypertension, diabetes, hyperlipidemia, cardiac dysfunction, atrial fibrillation, or > mild valvular disease); (ii) smoking; (iii) current use of cardiorespiratory medications, chemotherapeutic agents, or vasoactive drugs; (iv) hepatopulmonary syndrome or pulmonary arteriovenous malformations; and (v) suboptimal echocardiographic image quality per standard guidelines.

### Data collection

Demographics, residential altitude, and partial pressure of inspired oxygen (PiO_2_) were analyzed using retrospective data from participants, combined with records provided by the local meteorological administration and atmospheric research institute.

### Conventional transthoracic echocardiography

All transthoracic echocardiography examinations were performed by two experienced echocardiographer (with 20 years of clinical experience in echocardiography), using a commercially available ultrasound system (Philips EPIQ 7C, Netherlands) equipped with an X5-1 PureWave xMATRIX transducer (1–5MHz). According to the recent EACVI/ASE recommendations[8,9], the following parameters were obtained: left ventricular end-diastolic anteroposterior diameter (D_LV_), right ventricular end-diastolic anteroposterior diameter (D_RV_), left atrial end-systolic transverse diameter (D_LA_), right atrial end-systolic transverse diameter (D_RA_), left ventricular ejection fraction (LVEF), left ventricular mass index (LVM index), the ratio of the peak early diastolic transmitral filling velocity to the peak early diastolic lateral mitral annulus tissue velocity (E/e’), tricuspid annular plane systolic excursion (TAPSE), the peak tricuspid regurgitation velocity (TR_Vmax_). Finally, estimated PASP was calculated using the formula: PASP (mmHg) = 4TR_Vmax_^2^ + right atrial pressure.

### pPTT measurement

**Fig. 1:**
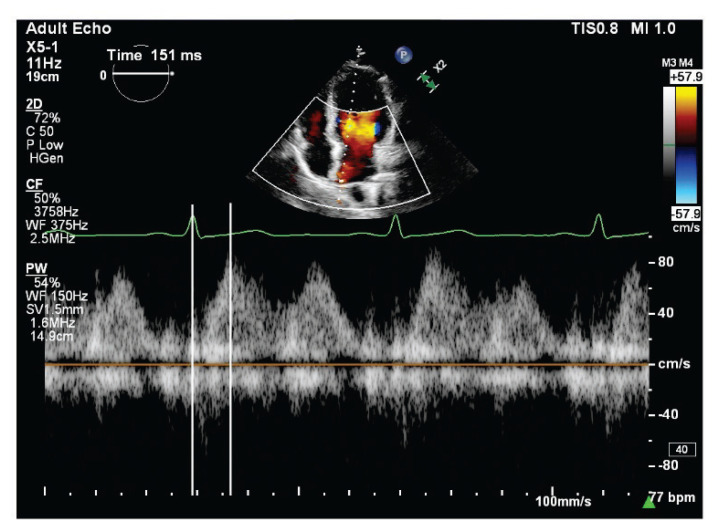
Pulmonary vein flow pulsed-wave Doppler tracing in a high-altitude resident, illustrating the measurement methodology of pulmonary pulse transit time (pPTT, ms) in this study. pPTT was defined as the time interval (ms) between the R-wave in the electrocardiogram and the peak late systolic pulmonary vein flow velocity (R-PVs2 interval).

In our research, pPTT was measured using the right superior pulmonary vein visualized from the apical four-chamber view. By optimizing transducer positioning, we achieved clear visualization of the pulmonary vein orifice. To minimize artifacts, the gain was carefully adjusted to enhance image contrast. A pulsed-wave Doppler sample volume of 2–3 mm was positioned at the pulmonary vein opening, with a moderate sweep speed of 75 mm/s. The velocity scale was initially set between 20–40 cm/s to capture low-velocity blood flow signals, with adjustments made as needed. A low wall filter setting was maintained to reduce interference from the slow motion of the posterior atrial wall during pulmonary vein recording. Typically, in healthy adults, the pulmonary vein Doppler spectrum demonstrates three distinct waveforms: peak late systolic (S2), peak diastolic (D), and atrial reversal (A) velocities. The pPTT was specifically measured as the time interval from the electrocardiogram R-wave to the peak of the late systolic pulmonary venous flow (R-PVs2 interval) (Fig. 1), as described in previous studies [1,3,5,6]. For each measurement, we analyzed a minimum of three consecutive cardiac cycles and calculated the average value.

### Statistical analysis

The distribution of continuous variables was evaluated using the Shapiro-Wilk test. Normally distributed data were expressed as mean ± standard deviation (SD) and compared using independent samples t-tests. For variables violating normality assumptions (confirmed by both Shapiro-Wilk tests and Q-Q plot visualization), non-parametric analyses were employed, with data presented as median (IQR) and compared using Mann-Whitney U tests. Categorical variables were summarized as counts (percentages) and analyzed with chi-square tests. To determine independent predictors of pPTT, we performed multiple linear regression incorporating all demographic, clinical, and echocardiographic parameters that showed significant differences between high-altitude and low-altitude residents. Model assumptions were verified by: (1) assessing linearity via scatterplots, (2) checking multicollinearity (VIF <5 threshold), and (3) examining residual normality with Shapiro-Wilk testing. A two-tailed P-value <0.05 defined statistical significance. All analyses were conducted using SPSS 19.0 (IBM Corp.) and MedCalc 16.8.4 (MedCalc Software), with graphical assessments performed in both platforms.

## Results

### Characteristics of included patients

**Fig. 2:**
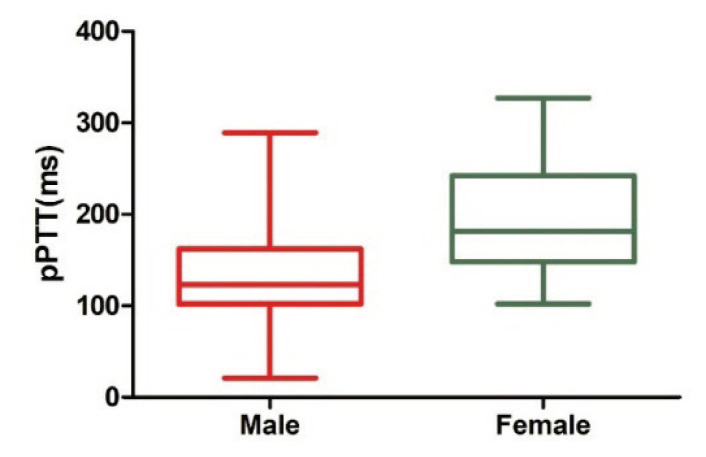
Comparison of pulmonary pulse transit time (pPTT, ms) between male and female high-altitude residents.

Between October 2024 and June 2025, 47 of the 120 high-altitude residents were excluded due to pulmonary arterial hypertension (PASP ≥30 mmHg), 4 were excluded due to hypertension, and 22 were excluded due to smoking. Ultimately, 47 individuals were included in the study (31 males and 16 females). In both the high-altitude and low-altitude residents, more than 85 % of participants were engaged in farming. As shown in Table 1, the altitude was significantly higher and the PiO_2_ level was significantly lower in the high-altitude region compared to the low-altitude region (*p* < 0.0001). High-altitude residents showed no significant differences in age, gender, BSA, body mass index (BMI), heart rate, systolic and diastolic blood pressure compared to low-altitude residents (*p* > 0.05). However, high-altitude residents exhibited significantly larger D_RV_, D_RA_ and TAPSE, but significantly lower LVM index and LVEF than low-altitude residents (*p* < 0.05). Notably, pPTT was markedly shorter in high-altitude residents compared to their low-altitude counterparts (*p* < 0.0001). Furthermore, among high-altitude residents, males had significantly shorter pPTT than females (123.00 [102.00–162.00] ms vs. 181.50 [148.00–241.00] ms, *p* < 0.0001) (Fig. 2). No significant differences were observed in other echocardiographic parameters (*p* > 0.05).

### Multiple linear regression analysis

Whether in the enter or stepwise multiple regression analysis, only altitude was the independent determinant of pPTT in high-altitude residents (*p* <0.05) (Table 2 and Table 3). The regression equation of this optimal model generated from these determinants was pPTT = 0.314×Altitude - 1119.698 (R = 0.236; R^2^ = 0.056; R^2^-adjusted = 0.048; F = 7.362; and *p* <0.008).

### Intra-/interobserver variability

To assess measurement reliability, intraobserver and interobserver variability analyses were conducted. For intraobserver consistency, Observer A repeated pPTT measurements in 30 randomly selected cases after an 8-week interval to reduce recall bias, demonstrating exceptional reproducibility with an intraclass correlation coefficient (ICC) of 0.98 (95 % confidence interval [CI]: 0.98–0.99) and a coefficient of variation (CV) of 2.3 %. Interobserver agreement was evaluated by having two independent observers (Observer A and B) analyze the same 30 cases, revealing strong concordance with an ICC of 0.97 (95 % CI: 0.92–0.98) and a CV of 2.9 %. These results confirm high measurement consistency both within and between observers.

## Discussion

This study systematically demonstrates for the first time a significant shortening of pPTT in healthy high-altitude residents without pulmonary hypertension. This pivotal finding carries multiple scientific implications: First, it indicates that prolonged high-altitude hypoxic exposure can induce characteristic functional alterations in the pulmonary arterial system even when pulmonary artery pressure remains normal. Second, the temporal pattern showing pPTT changes preceding significant variations in conventional hemodynamic parameters (e.g., PASP) strongly suggests that pPTT may serve as a more sensitive indicator for assessing pulmonary vascular function. Particularly noteworthy is our additional finding of significantly shorter pPTT in male participants compared to females.

It is well known that pulse wave transit time is inversely related to pulse wave velocity. The severity of pulmonary stiffness is directly correlated with pulse wave velocity-the stiffer the pulmonary arteries, the faster the pulse wave velocity and consequently the shorter the pPTT. In our study, potential mechanisms underlying the shortening of pPTT may include the following aspects: (i) Chronic hypoxia can induce significant structural remodeling of the pulmonary arteries through the activation of signaling pathways such as Rab26 [10], leading to increased intrinsic stiffness of the pulmonary vasculature. Histological studies have demonstrated that high-altitude residents exhibit increased medial thickness and disorganized elastic fiber arrangement in the pulmonary arteries [11,12]. Additionally, there is enhanced muscularization of distal pulmonary arteries, with smooth muscle cell extension into previously non-muscularized small arterioles [13], leading to increased functional stiffness of the pulmonary vasculature. Our study found that right atrial and ventricular enlargement may be associated with pulmonary vasoconstriction and reduced pulmonary vascular bed capacity. (ii) For individuals with acute high-altitude exposure, the shortened pPTT may primarily reflect a functional elevation in vascular tension due to hypoxic pulmonary vasoconstriction [14,15]. This functional plasticity is further illustrated by a study involving 21 participants, which demonstrated that systemic pulse wave velocity progressively increased with ascending altitude (corresponding to a shortened pulse wave transit time), but returned to or even fell below baseline levels upon descent to low altitude [15]. These findings confirm that the changes in pulse wave transit time are highly reversible after acute exposure, with the potential for supernormal functional recovery. However, for multi-generational residents like our study cohort, long-term hypoxic exposure is likely to have induced epigenetic and even structural adaptations. Consequently, the vascular stiffness represented by their shortened pulse wave transit time is probably more fixed, with a relatively limited potential for reversibility. Future longitudinal studies tracking pulse wave transit time in lowlanders before and after migration to high altitude would be ideal for elucidating this dynamic process of adaptation and de-adaptation. (iii) Furthermore, the potential association between pPTT and hemorheological parameters (such as hematocrit and blood viscosity) warrants further investigation. The prevalent compensatory erythrocytosis in high-altitude natives and the consequent increase in blood viscosity can theoretically contribute to regulating the pulmonary circulatory load by increasing flow resistance and vascular wall shear stress [16,17]. Although limited by its design, our study did not concurrently measure hematocrit or other parameters to directly verify their correlation with pPTT. However, it is plausible that a synergistic or covariant relationship exists between them during chronic hypoxic adaptation. We speculate that the shortened pPTT in high-altitude residents results from the combined effects of the direct remodeling impact of chronic hypoxia on the pulmonary vasculature and the indirect hemodynamic consequences of altered hemorheology. Clarifying the relationship between pPTT and hematocrit will help distinguish the relative contributions of intrinsic vascular properties versus blood-related factors in high-altitude pulmonary vascular adaptation in future research, which is undoubtedly a crucial direction.

The observed gender disparities in pPTT may result from multiple factors: testosterone promotes vascular smooth muscle proliferation while estrogen enhances endothelium-dependent vasodilation [18–20]; males generally have larger body surface area and longer vascular pathways; higher hemoglobin concentrations and increased blood viscosity in males; and typically elevated sympathetic tone in males [21]. These physiological differences collectively contribute to the shorter pPTT observed in male high-altitude residents.

While prior studies directly measuring pulmonary arterial compliance or pulse wave velocity (or pulse wave transit time) via invasive catheterization or magnetic resonance imaging (MRI) specifically in healthy high-altitude residents are lacking, these established methodologies have provided foundational insights from different perspectives. Invasive cardiac catheterization, the gold standard for measuring pulmonary arterial pressure and resistance, has directly documented hypoxic pulmonary vasoconstriction and increased vascular resistance at high altitude. However, its invasive nature precludes its use for large-scale screening or longitudinal monitoring in healthy populations. Conversely, MRI allows for non-invasive and precise assessment of main pulmonary artery distensibility and hemodynamics [22–24], serving as a powerful tool for evaluating large vessel properties, yet its high cost and limited availability restrict its practical application in remote high-altitude settings. The pPTT, as a non-invasive, simple, and cost-effective echocardiographic derivative used in our study, directly reflects increased global stiffness of the pulmonary arterial tree. Our findings provide direct functional evidence from a healthy population that corroborates the decreased pulmonary vascular compliance and structural remodeling previously suggested indirectly by invasive and MRI studies. pPTT thus holds promise as a bridge connecting traditional hemodynamic measurements with the detection of early vascular dysfunction.

The pPTT and the widely recommended indicator of right ventricular-pulmonary arterial (RV-PA) coupling—the TAPSE/PASP ratio—offer distinct yet complementary perspectives on the right heart-pulmonary artery system. The TAPSE/PASP ratio is valued for its integrative assessment of the efficiency of coupling between right ventricular systolic function (TAPSE) and its afterload (PASP), serving as an excellent indicator of ventricular-afterload coupling [2,25]. However, in early disease stages, right ventricular function may be maintained through compensatory mechanisms, potentially limiting this ratio’s sensitivity to initial vascular pathology. In contrast, pPTT provides a unique vessel-centric viewpoint. It directly reflects the intrinsic physical properties (stiffness) of the pulmonary arterial wall itself. A key finding of our study is that pPTT was significantly shortened in healthy high-altitude residents even in the absence of significantly elevated PASP (i.e., without a substantial increase in afterload). This strongly suggests that pPTT can detect early vascular functional remodeling that precedes a measurable rise in afterload. Therefore, the combined application of pPTT (assessing vascular properties) and the TAPSE/PASP ratio (assessing ventricular-afterload coupling) enables a more comprehensive and earlier non-invasive evaluation of the RV-PA system. This multi-parameter strategy holds future potential for more precise risk stratification of the transition from physiological adaptation to pathological pulmonary hypertension in high-altitude populations.

This study has several limitations that should be acknowledged. First, the relatively small final cohort size may limit statistical power. Second, while altitude was a significant independent predictor in our regression model for pPTT, it explained only a modest proportion of the total variance (adjusted R^2^ = 0.048), indicating that other unmeasured genetic, hemodynamic, or environmental factors play a major role. Third, a total of 73 high-altitude residents were excluded from the initial 120: 47 for pulmonary arterial hypertension, 4 for hypertension, and 22 for smoking. The stringent exclusion criteria, while necessary to isolate a healthy cohort, may introduce selection bias and limit the generalizability of our findings to the broader high-altitude population. Finally, the absence of hematocrit and blood viscosity measurements in our study design precludes a direct analysis of the correlation between pPTT and these key hemorheological parameters. Future studies with larger sample sizes, incorporation of comprehensive biomarker profiling are warranted to validate and extend our findings.

## Conclusion

This study demonstrates that healthy high-altitude residents exhibit significantly shortened pPTT independent of pulmonary hypertension, reflecting chronic hypoxia-induced adaptive vascular remodeling. As a more sensitive indicator than traditional hemodynamic parameters, pPTT shows promise as a non-invasive biomarker for monitoring pulmonary vascular status. Future longitudinal studies are warranted to validate its predictive value for high-altitude pulmonary hypertension and elucidate the underlying mechanisms of observed sex differences.

## Figures and Tables

**Table 1:** Descriptive demographic, clinical, and echocardiographic characteristics of all participants

Variables	Low-altitude residents(n=50)	High-altitude residents (n=47)	P value
Altitude*, m*	3.70(2.08–4.35)	4050.00(4010.00–4064.00)	<0.0001
*PiO* *_2_* *, kPa*	21.20(21.20–21.21)	12.79(11.53–12.82)	<0.0001
*Farmers, case (%)*	43(86.00)	41(87.23)	
*Age, years*	32.50(26.50–43.25)	35.00(29.00–41.00)	
*Male, case (%)*	30(60.00)	31(65.96)	
*BSA, m2*	1.72(1.54–1.92)	1.74(1.62–1.86)	
*BMI,kg/m* *^2^*	23.36(20.86 – 25.96)	23.03(20.50 –25.76)	
*Heart rate,bpm*	72.50(63.13–79.00)	67.00(63.00–75.25)	0.086
*SBP, mmHg*	121.00(110.00–139.00)	124.00(115.00–134.00)	
*DBP, mmHg*	86.00(75.00–95.00)	85.50(78.00–91.00)	
*D* *_RV_* *, mm*	19.00(18.00–20.00)	21.00(22.00–25.00)	<0.0001
*D* *_RA_* *, mm*	31.00(30.00–34.00)	33.00(31.00–36.00)	0.0002
*D* *_LV_* *, mm*	45.00(42.00–47.00)	45.00(43.00–47.00)	
*D* *_LA_* *, mm*	31.00(30.00–33.50)	31.00(28.00–33.00)	
*LVM index, g/m* *^2^*	76.38(69.44–81.02)	72.62(63.27–78.06)	0.0012
*LVEF, %*	69.00(66.00–72.00)	65.00(62.00–68.00)	<0.0001
*Mitral E/e’*	6.49(4.41–7.82)	6.30(5.19–6.92)	
*TAPSE, mm*	20.50(20.00–23.00)	21.00(18.00–22.00)	0.0124
*PASP, mmHg*	18.00(15.00–24.00)	17.00(13.00–25.00)	
*TAPSE/PASP ratio,*	1.00(0.91–1.18)	1.06(0.82–1.64)	
*mm/mmHg, pPTT, ms*	193.50(179.00–285.00)	144.00(109.00–193.00)	<0.0001

Data are expressed as number (percentage) or median (interquartile range). BSA, body surface area; BMI, body mass index; bpm, beats per minute; D_LA_, left atrial end-systolic anteroposterior diameter; DBP, diastolic blood pressure; D_LV_, left ventricular end-diastolic anteroposterior diameter; D_RA_, right atrial end-systolic transverse diameter; D_RV_, right ventricular end-diastolic anteroposterior diameter; E/e’, the ratio of peak early diastolic transmitral filling velocity (E) and peak early diastolic lateral mitral annulus tissue velocity(e’); LVEF, left ventricular ejection fraction; LVM, left ventricular mass; PASP, pulmonary artery systolic pressure; PiO_2_, partial pressure of inspired oxygen; pPTT, pulmonary pulse wave transit time; SBP, systolic blood pressure; TAPSE, tricuspid annular plane systolic excursion.

**Table 2:** Enter multiple linear regression analysis for pPTT and related parameters

Independent variables	Unstandardized Coefficients		Standardized Coefficients	t	Sig
	B	Std.Error	Beta		
Altitude	0.314	0.116	0.236	2.707	0.008
PiO_2_	−1.197	1.042	−0.103	−1.148	0.253
DRV,	−0.967	2.042	−0.042	−0.473	0.637
DRA,	−1.045	1.415	−0.066	−0.738	0.462
LVM index	0.230	0.424	0.049	0.543	0.588
LVEF	2.435	1.492	0.145	1.632	0.105
TAPSE	1.158	2.674	0.039	0.433	0.666

DRA, right atrial end-systolic transverse diameter; DRV, right ventricular end-diastolic anteroposterior diameter; LVEF, left ventricular ejection fraction; LVM, left ventricular mass; pPTT, pulmonary pulse wave transit time; PiO_2_, partial pressure of inspired oxygen; TAPSE, tricuspid annular plane systolic excursion.

**Table 3:** Stepwise multiple linear regression analysis for pPTT and related parameters

Independent variables	Unstandardized Coefficients		Standardized Coefficients	t	Sig	VIF
	B	Std.Error	Beta			
(Constant)	−1119.698	472.288	−0.193	−2.371	0.019	
Altitude	0.314	0.116	0.236	2.707	0.008	1.000

pPTT, pulmonary pulse wave transit time; VIF, variance inflation factor.
